# Corrigendum: Plasma Markers of Disrupted Gut Permeability in Severe COVID-19 Patients

**DOI:** 10.3389/fimmu.2021.779064

**Published:** 2021-10-04

**Authors:** Leila B. Giron, Harsh Dweep, Xiangfan Yin, Han Wang, Mohammad Damra, Aaron R. Goldman, Nicole Gorman, Clovis S. Palmer, Hsin-Yao Tang, Maliha W. Shaikh, Christopher B. Forsyth, Robert A. Balk, Netanel F. Zilberstein, Qin Liu, Andrew Kossenkov, Ali Keshavarzian, Alan Landay, Mohamed Abdel-Mohsen

**Affiliations:** ^1^ The Wistar Institute, Philadelphia, PA, United States; ^2^ The Burnet Institute, Melbourne, VIC, Australia; ^3^ Department of Infectious Diseases, Monash University, Melbourne, VIC, Australia; ^4^ Rush Center for Integrated Microbiome and Chronobiology Research, Rush University, Chicago, IL, United States; ^5^ Department of Internal Medicine, Rush University Medical Center, Chicago, IL, United States

**Keywords:** SARS-CoV-2, COVID-19, microbial translocation, inflammation, zonulin, metabolomics, glycomics, lipidomics

In the original article, there was a typo in **Figure 1E**, **Supplementary Figure 3**, and **Supplementary Table 10** as published. The unit of the β-glucan should have been (pg/ml) instead of (ng/ml). The corrected **Figure 1**, **Supplementary Figure 3**, and **Supplementary Table 10** appear below.

**Figure 1:**
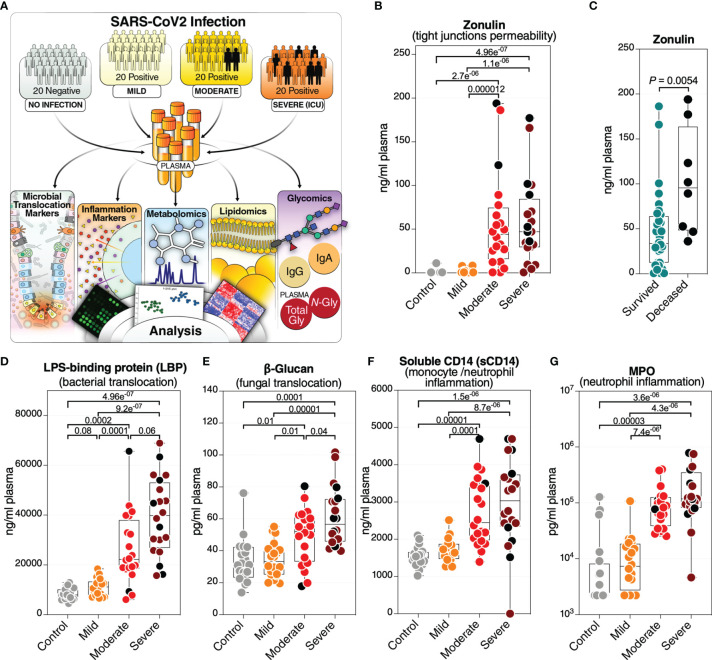
Severe COVID-19 is associated with an increase in markers of tight junction permeability and microbial translocation. **(A)** An overview of the main cohort study design; moderate and severe patients were hospitalized; severe indicates patients in the intensive care unit. **(B)** Levels of plasma zonulin, are higher during moderate and severe COVID-19 compared to mild COVID-19 or controls. Kruskal–Wallis test was used for statistical analysis. False discovery rate (FDR) was calculated using the Benjamini-Hochberg method. Symbols in black indicate deceased. **(C)** Zonulin levels are higher in hospitalized COVID patients (n=40) who eventually died from COVID-19 (n=8) compared to survivors (n=32). Nominal P-value was calculated using the Mann–Whitney U test. **(D–G)** Levels of LBP **(D)**, β-Glucan **(E)**, sCD14 **(F)**, and MPO **(G)**, are higher during severe COVID-19 compared to mild COVID-19 or controls. Kruskal–Wallis test was used for statistical analysis. FDR was calculated using Benjamini-Hochberg method. Black dots indicate deceased.

The authors apologize for this error and state that this does not change any of the scientific conclusions of the article in any way. The original article has been updated.

## Publisher’s Note

All claims expressed in this article are solely those of the authors and do not necessarily represent those of their affiliated organizations, or those of the publisher, the editors and the reviewers. Any product that may be evaluated in this article, or claim that may be made by its manufacturer, is not guaranteed or endorsed by the publisher.

