# Supplementary material for: Plasma Markers of Disrupted Gut Permeability in Severe COVID-19 Patients
Source: Front Immunol. 2021 Jun 9;12:686240. doi: 10.3389/fimmu.2021.686240 (PMC8219958; doi:10.3389/fimmu.2021.686240)

Supplementary Figure 1

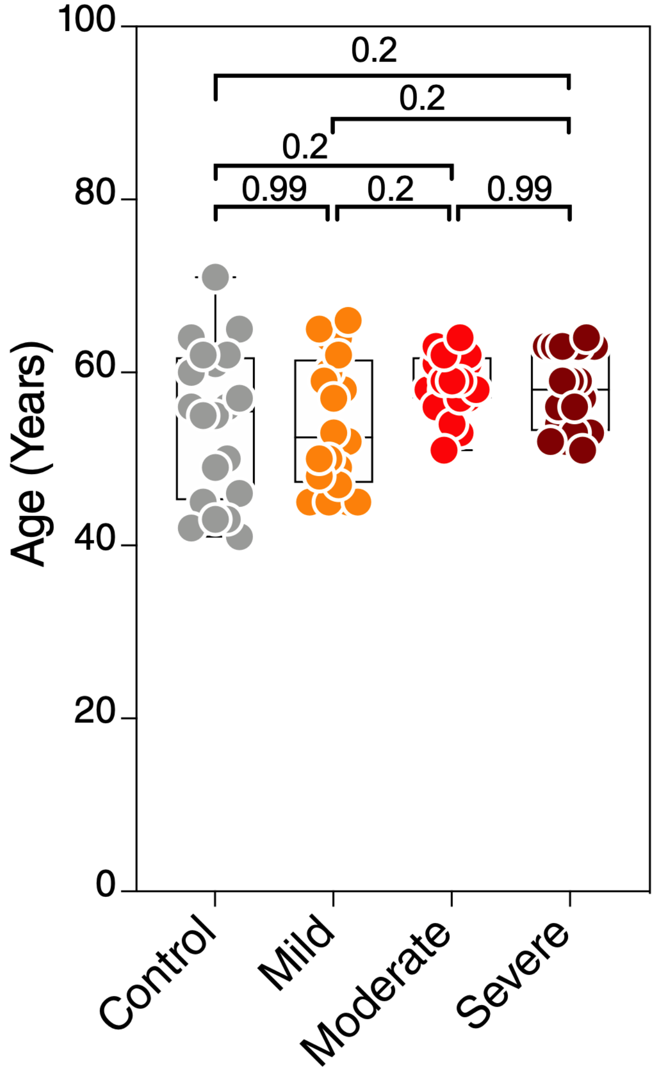

Supplementary Figure 2

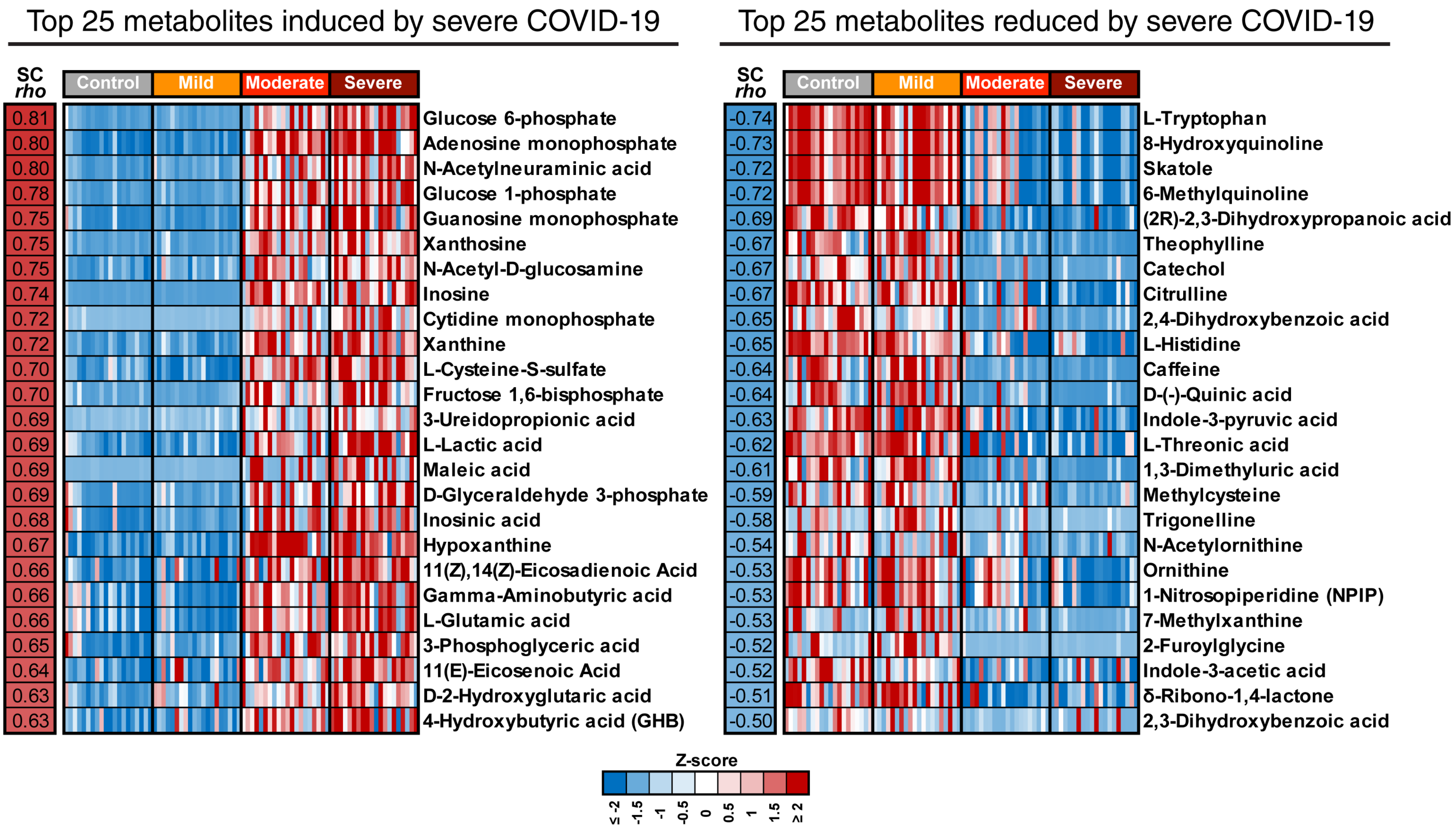

Supplementary Figure 3

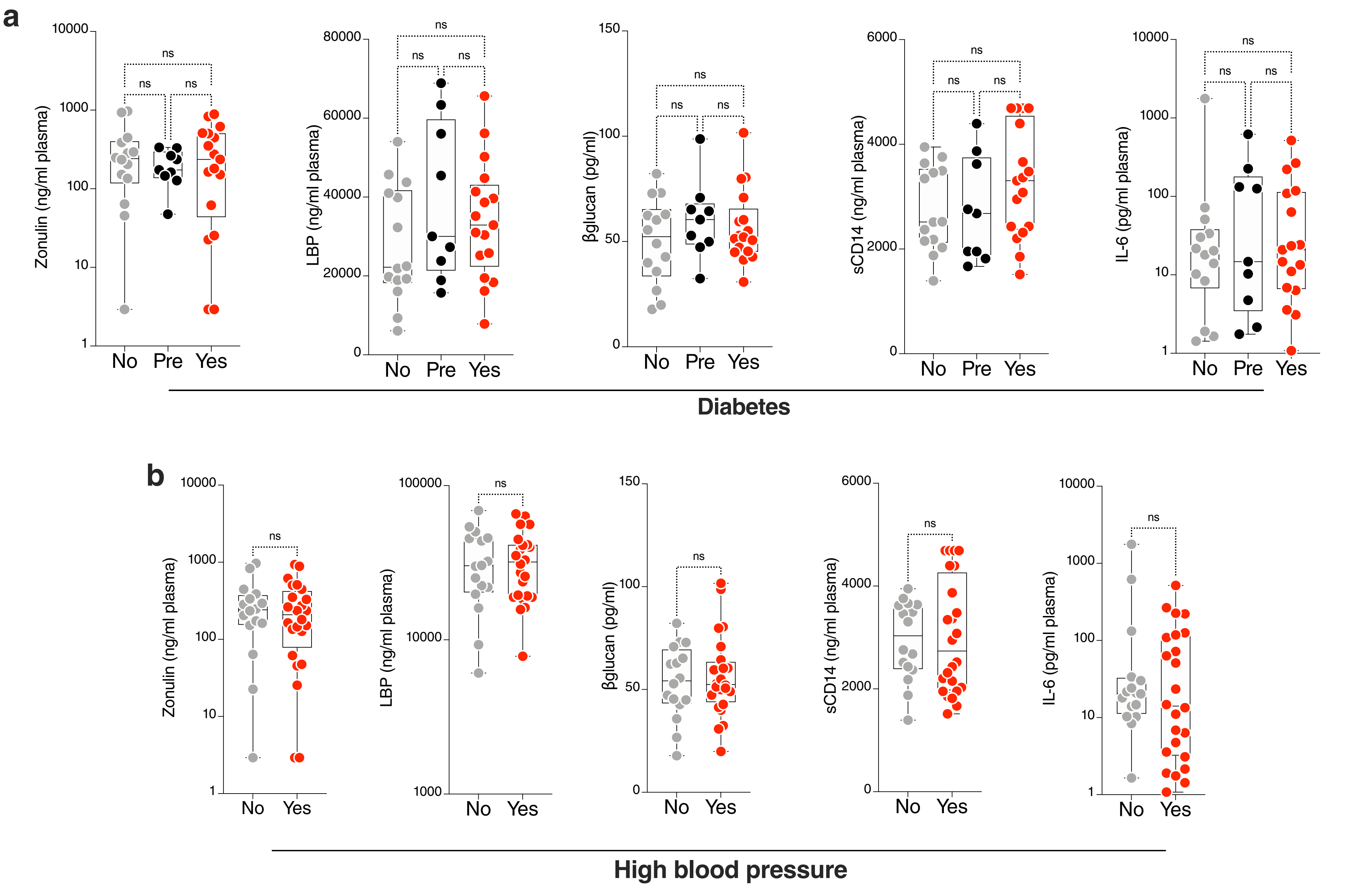

Supplementary Figure 4

a

Microbial Translocation

Glycome	SC rho	LBP	Zonulin	sCD14	βglucan	Occludin	sCD163
SC rho		0.81	0.77	0.74	0.60	0.31	0.25
TJAI-binding	0.74	0.66	0.57	0.56	0.42	0.21	0.11
TcLcl-binding	0.68	0.69	0.51	0.53	0.56	0.19	0.17
A2	0.62	0.64	0.61	0.49	0.53	0.14	0.18
Tetra-sialylated	0.59	0.66	0.57	0.52	0.67	0.34	0.31
A4G4S4	0.59	0.66	0.57	0.52	0.67	0.34	0.31
DSA-binding	0.52	0.45	0.33	0.40	0.51	0.10	0.11
Tetra-galactosylated	0.51	0.55	0.49	0.45	0.58	0.29	0.25
ConA-binding	0.49	0.48	0.38	0.34	0.55	0.10	0.13
Total sialylated	0.44	0.50	0.35	0.45	0.29	0.18	0.06
AOL-binding	0.41	0.40	0.41	0.37	0.32	0.20	0.14
Di-sialylated	0.41	0.53	0.32	0.48	0.35	0.20	0.00
ABA-binding	0.38	0.34	0.34	0.29	0.25	0.17	0.25
Di-sialylated	0.36	0.32	0.45	0.24	0.29	-0.02	0.02
High-branched	0.35	0.37	0.27	0.30	0.30	0.17	0.14
AsG2S2	0.35	0.52	0.34	0.48	0.41	0.22	0.03
A3F1G3S3	0.32	0.32	0.31	0.23	0.24	0.05	0.23
Branched Fucose	0.31	0.30	0.30	0.22	0.22	0.04	0.22
Fucose	-0.21	-0.25	-0.40	-0.26	-0.32	-0.18	-0.30
AsG2S2	-0.27	-0.31	-0.33	-0.28	-0.36	-0.28	-0.30
WFA-binding	-0.30	-0.19	-0.27	-0.31	-0.05	-0.09	-0.06
G1FB	-0.33	-0.31	-0.26	-0.12	-0.32	-0.12	-0.12
Core Fucose	-0.33	-0.47	-0.32	-0.48	-0.31	-0.20	-0.08
Terminal di-galactose	-0.35	-0.29	-0.26	-0.33	-0.22	-0.26	-0.22
Low-branched	-0.35	-0.37	-0.27	-0.30	-0.31	-0.17	-0.14
FA2G1	-0.35	-0.44	-0.29	-0.44	-0.30	-0.22	-0.06
G1F-6_arm	-0.40	-0.46	-0.45	-0.39	-0.37	-0.19	-0.44
G2F	-0.40	-0.32	-0.34	-0.39	-0.18	-0.24	-0.22
A3G3S2	-0.40	-0.45	-0.44	-0.40	-0.44	-0.22	-0.14
FA2BG1	-0.42	-0.51	-0.38	-0.34	-0.38	-0.09	-0.19
Non-sialylated	-0.44	-0.50	-0.35	-0.45	-0.29	-0.18	-0.06
Mono-sialylated	-0.45	-0.49	-0.39	-0.47	-0.41	-0.32	-0.10
Termial di-galactose	-0.46	-0.39	-0.40	-0.43	-0.24	-0.26	-0.28
NPA-binding	-0.47	-0.48	-0.37	-0.36	-0.31	-0.08	-0.05
VVA-bindning	-0.47	-0.42	-0.40	-0.49	-0.19	-0.28	-0.15
FA2G2S1	-0.47	-0.57	-0.41	-0.57	-0.45	-0.23	-0.16
Terminal mono-galactose	-0.49	-0.59	-0.39	-0.53	-0.51	-0.31	-0.06
G2FB	-0.52	-0.51	-0.49	-0.36	-0.42	-0.22	-0.36
FA2G2S1_FABG2S1	-0.52	-0.61	-0.48	-0.43	-0.50	-0.28	-0.23
M6	-0.52	-0.40	-0.47	-0.39	-0.34	-0.29	-0.26
FA2G2S1	-0.53	-0.67	-0.45	-0.48	-0.60	-0.09	-0.23
A2G2_FA2BG1	-0.53	-0.58	-0.42	-0.38	-0.40	-0.22	-0.11
A4G3S3	-0.53	-0.68	-0.51	-0.48	-0.58	-0.16	-0.15
FA2BG2	-0.54	-0.64	-0.48	-0.46	-0.53	-0.22	-0.23
FA2G1	-0.55	-0.64	-0.48	-0.60	-0.43	-0.24	-0.23
RCA120-binding	-0.56	-0.54	-0.49	-0.48	-0.44	-0.33	-0.10
Terminal galactose	-0.57	-0.67	-0.45	-0.59	-0.54	-0.35	-0.11
UDA-binding	-0.58	-0.63	-0.44	-0.43	-0.55	-0.24	-0.21
ACG-binding	-0.60	-0.62	-0.58	-0.55	-0.53	-0.26	-0.27
STL-binding	-0.60	-0.50	-0.43	-0.39	-0.46	-0.08	-0.06
FA2G2	-0.66	-0.73	-0.54	-0.70	-0.50	-0.30	-0.25
Terminal di-galactose	-0.69	-0.77	-0.57	-0.68	-0.56	-0.30	-0.25

Cross-correlations

b

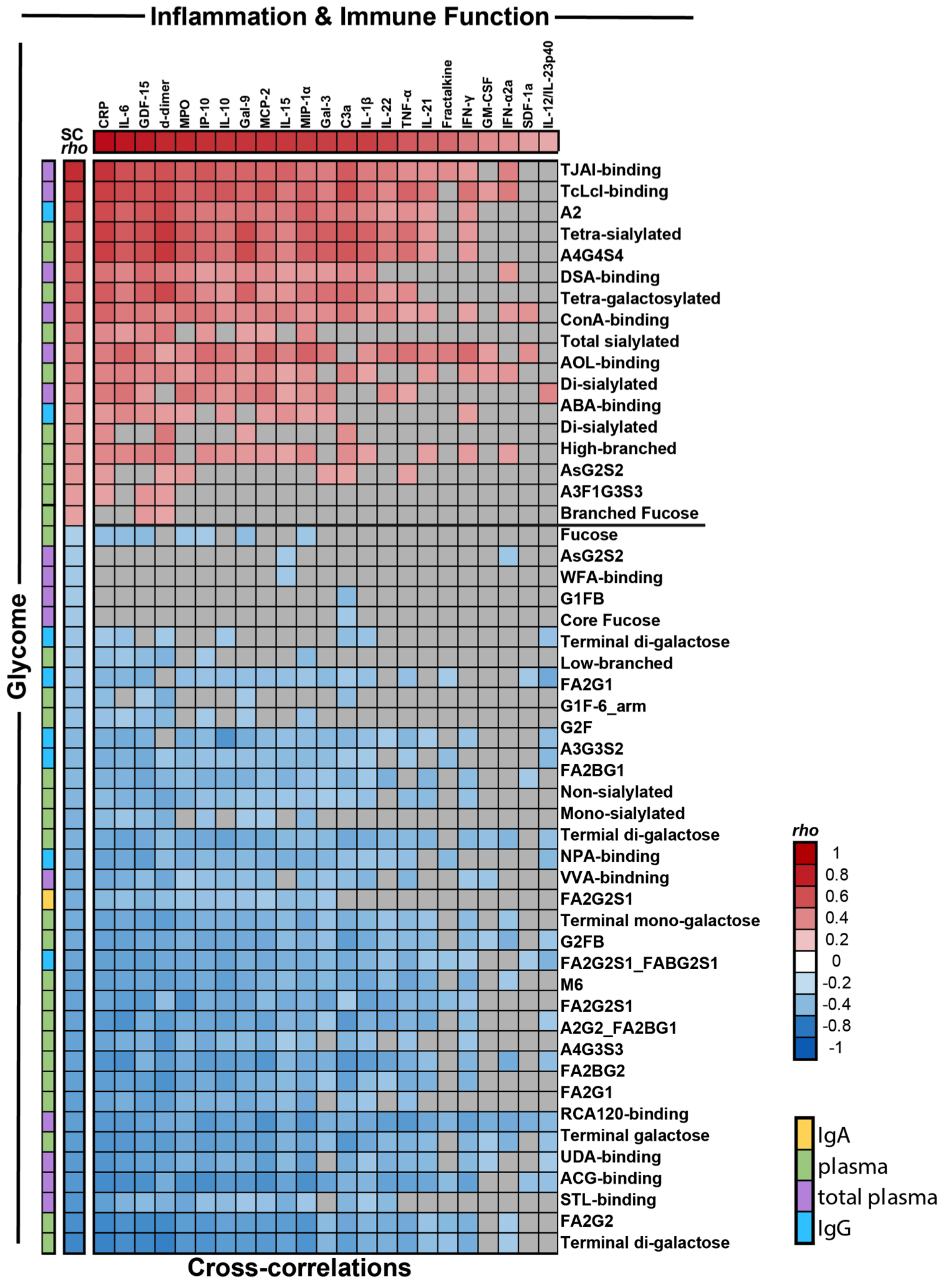

Supplement: Supplementary Figure 1 — Age was not significantly different between the groups in the main cohort. Kruskal–Wallis test was used for statistical analysis [file DataSheet_1.pdf]
