# Supplementary material for: Plasma Markers of Disrupted Gut Permeability in Severe COVID-19 Patients
Source: Front Immunol. 2021 Jun 9;12:686240. doi: 10.3389/fimmu.2021.686240 (PMC8219958; doi:10.3389/fimmu.2021.686240)
Supplement: Supplementary file 2 [file DataSheet_2.pdf]

Supplementary Table 1. Demographic and clinical characteristics of the main study cohort.

		SARS-CoV-2 Negative -	SARS-CoV-2 Positive Mild (outpatients)	SARS-CoV-2 Positive Moderate (Inpatients)	SARS-CoV-2 Positive Severe (ICU)
Number *(n)*		20	20	20	20
Female, *n* (%)		10 (50)	12 (60)	11 (55)	7 (35)
Age, years, median (IQR)		55.5 (15.6)	52.5 (12.75)	58.5 (4.25)	58 (9.25)
Deceased, *n* (%)		0 (0)	0 (0)	2 (10)	6 (30)
Body mass index (BMI)					
	Normal Weight (<25), %	-	6.7*	25	5
	Overweight/obese (>25), %	-	93.3*	75	95
Pre-diabetes, %		-	6.7*	15	30
Diabetes Mellitus (DM), %		-	6.7*	30	55
High blood pressure, %		-	33.3*	60	60
Asthma,%		-	13.3*	15	5
Hydroxychloroquine, %		-	6.7*	40	55
Remdesivir, %		-	-	5	25
Tocilizumab, %		-	-	5	30
Chronic steroid use, %		-	-	25	5
Acute steroid use, %		-	-	15	45
Plasma IV nutrition, %		-	-	0	0
Enteral nutrition use, %		-	-	100	100
Antibiotic administration, %		-	-	60	85
Ethnicity		-	-		
	African American, *n* (%)	0	0	9 (45)	7 (35)
	Hispanic or Latino, *n* (%)	0	2 (10)	6 (30)	9 (45)
	Caucasian, *n* (%)	3 (15)	13 (65)	4 (20)	3 (15)
	Other, *n* (%)	2 (10)	1 (5)	0	1 (5)
	Unknown, *n* (%)	15 (75)	4 (20)	1 (5)	0

*Based on 15 out of 20 donors with available data

Supplementary Table 2. Demographic characteristics of the validation cohort.

		SARS-CoV-2 Negative<br>-	SARS-CoV-2 Positive<br>Mild (outpatients)	SARS-CoV-2 Positive<br>Moderate (Inpatients)	SARS-CoV-2 Positive<br>Severe (ICU)
Number *(n)*		18	20	18	19
Female, *n* (%)		12 (67)	9 (45)	6 (33)	5 (26)
Age, years, median (IQR)		38.5 (19.25)	60 (10.5)	64.5 (14.5)	63 (15.5)
Ethnicity		-	-		
	African American, *n* (%)	0 (0)	3 (15)	6 (33.3)	2 (10.5)
	Hispanic or Latino, *n* (%)	1 (5.6)	6 (30)	9 (50)	11 (57.9)
	Caucasian, *n* (%)	15 (83.3)	5 (25)	3 (16.7)	5 (26.3)
	Other, *n* (%)	2 (11.1)	3 (15)	0 (0)	1 (5.3)
	Unknown, *n* (%)	0 (0)	3 (15)	0 (0)	0 (0)

Supplementary Table 3. The structures and names of *N*-glycans identified in plasma by capillary electrophoresis.

Glycan name and structure	Group	Glycan name and structure	Group
A2F1G2S2 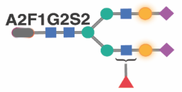	S2 + ST + FB + LB	FA2BG2 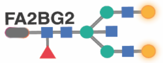	G2 + GT + B + FC + LB
A2G2 + FA2BG1 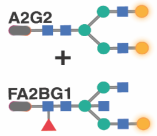	G2 + GT + LB	FA2BG2S2 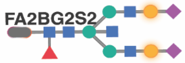	S2 + ST + B + FC + LB
A2G2S1 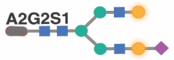	S1 + ST + LB	FA2G0 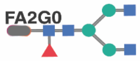	G0 + FC + LB
A2G2S1 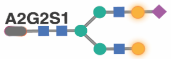	S1 +ST + G1 + GT + LB	FA2G1 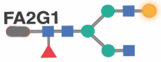	G1 + GT + FC + LB
A2G2S2 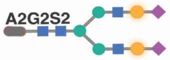	S2 + ST + LB	FA2G1 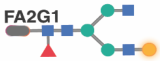	G1 + GT + FC + LB
A3F1G3S3 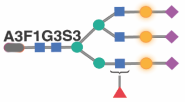	S3 + ST + FB + HB	FA2G2 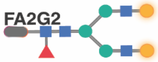	G2 + GT + FC + LB
A3G3S2 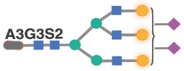	S2 + ST + G1 + GT + HB	FA2G2S1 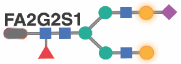	S1 + ST + G1 + GT + FC + LB
A3G3S3 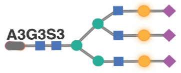	S3 + ST + HB	FA2G2S1 + FA2BG2S1 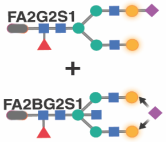	S1 + ST + G1 + GT + FC + LB
A4G4S3 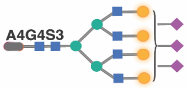	S3 + ST + G1 + GT + HB	FA2G2S1 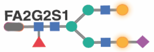	S1 + ST + G1 + GT + FC + LB
A4G4S4 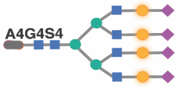	S4 + ST + HB	FA2G2S2 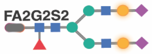	S2 + ST + FC + LB
FA2BG0 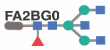	G0 + B + FC+ LB	FA3G3S2 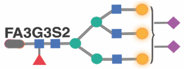	S3 + ST + G1 + GT + FC + HB
FA2BG1 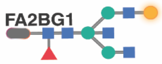	G1 + GT + B + FC+ LB	M6 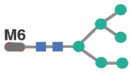	LB

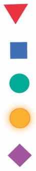

Fucose
N-acetylglucosamine
Mannose
Galactose
N-acetylneuraminic acid

These 24 glycan structures can be grouped into 15 groups: bisecting GlcNAc (B group), sialic acid (non-sialylated (S0), mono-sialylated (S1), di-sialylated (S2), tri-sialylated and (S3), tetra-sialylated (S4), and total sialylated (ST)), galactose (agalactosylated (G0), mono-galactosylated (G1), di-galactosylated (G2), and total galactosylated (GT)), core fucose (FC group), branched fucose (FB group), high branched (HB group), and low branch (LB group).

Supplementary Table 4. The structures and names of *N*-glycans identified in isolated IgG by capillary electrophoresis.

Glycan name and structure	Group	Glycan name and structure	Group
A2 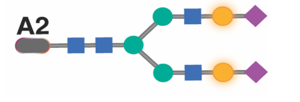	S2 + ST	G0FB 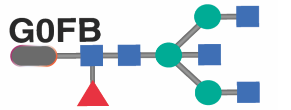	G0 + F + B
A2F 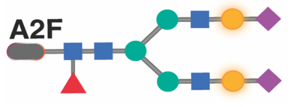	S2 + ST + F	G1F [6] 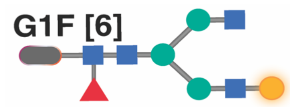	G1 + GT + F
A2FB 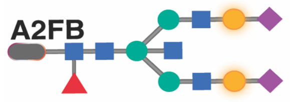	S2 + ST + F + B	G1F [3] 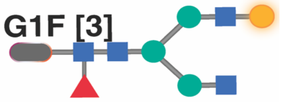	G1 + GT + F
A2B 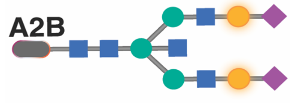	S2 + ST + B	G1FB 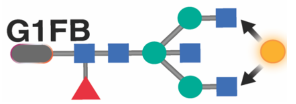	G1 + GT + F + B
G1FS1 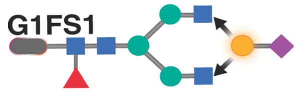	S1 + ST +F	G0 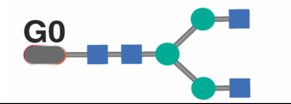	G0
A1 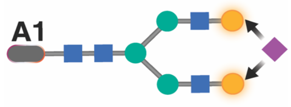	S1 + ST	G1S1 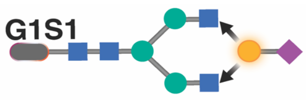	S1 + ST
A1F 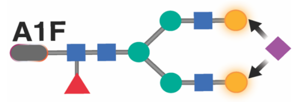	S1 + ST + F	G2 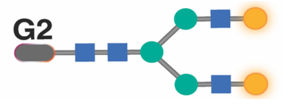	G2 + GT
A1FB 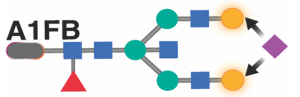	S1 + ST + F	G2F 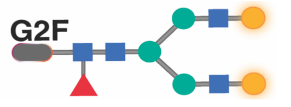	G2 + GT + F
G0F 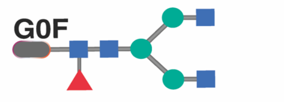	G0 + F	G2FB 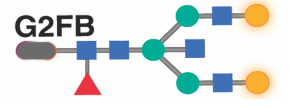	G2 + GT + F + B
G1 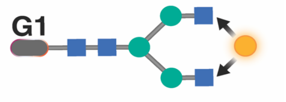	G1 + GT	G1B [3] 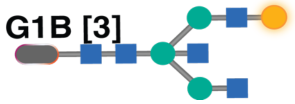	G1 + GT + B
G0B 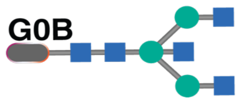	G0 + B	G1B [6] 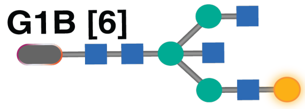	G1 + GT + B

Fucose
N-acetylglucosamine
Mannose
Galactose
N-acetylneuraminic acid

These 22 glycan structures were grouped into 9 groups, depending on the presence or absence of four key monosaccharides: bisecting GlcNAc (B group), sialic acid (mon-sialylated (S1), di-sialylated (S2), and total sialylated (ST)), terminal galactose (agalactosylated (G0), mono-galactosylated (G1), di-galactosylated (G2), and total galactose (GT)), and fucose (F group).

Supplementary Table 5. Lectins used for 45 lectin microarray.[1]

	Name	Species	Origin	Glycan specificity [2]
1	LTL	*Lotus tetragonolobus*	Natural	Fuc (Le^x, Le^y)
2	PSA	*Pisum sativum*	Natural	α1-6Fuc up to biantenna
3	LCA	*Lens culinaris*	Natural	α1-6Fuc up to biantenna
4	UEAI	*Ulex europaeus*	Natural	α1-2Fuc
5	AOL	*Aspergillus oryzae*	Recombinant	α1-6Fuc (Core), α1-2Fuc (H), α1-3Fuc (Le^x), α1-3Fuc (Le^a)
6	AAL	*Aleuria aurantia*	Natural	α1-6Fuc (Core), α1-2Fuc (H), α1-3Fuc (Le^x), α1-3Fuc (Le^a)
7	MAL	*Maackia amurensis*	Natural	α2-3Sia
8	SNA	*Sambucus nigra*	Natural	α2-6Sia
9	SSA	*Sambucus sieboldiana*	Natural	α2-6Sia
10	TJAI	*Trichosanthes japonica*	Natural	α2-6Sia
11	PHAL	*Phaseolus vulgaris*	Natural	GlcNAcβ1-6Man (Tetraantenna)
12	ECA	*Erythrina cristagalli*	Natural	βGal
13	RCA120	*Ricinus communis*	Natural	βGal
14	PHAE	*Phaseolus vulgaris*	Natural	bisecting GlcNAc
15	DSA	*Datura stramonium*	Natural	GlcNAcβ1-6Man (Tetraantenna)
16	GSLII	*Griffonia simplicifolia*	Natural	GlcNAcβ1-4Man
17	NPA	*Narcissus pseudonarcissus*	Natural	Manα1-3Man
18	ConA	*Canavalia ensiformis*	Natural	M3, Manα1-2Manα1-3(Manα1-6)Man, GlcNAcβ1-2Manα1-3(Manα1-6)Man
19	GNA	*Galanthus nivalis*	Natural	Manα1-3Man, Manα1-6Man
20	HHL	*Hippeastrum hybrid*	Natural	Manα1-3Man, Manα1-6Man
21	ACG	*Agrocybe cylindracea*	Natural	α2-3Sia
22	TxLcI	*Tulipa gesneriana*	Natural	Mannose/GalNac
23	BPL	*Bauhinia purpurea alba*	Natural	Galβ1-3GlcNAc(GalNAc), α/βGalNAc
24	TJAII	*Trichosanthes japonica*	Natural	α1-2Fuc
25	EEL	*Euonymus europaeus*	Natural	αGal (B)
26	ABA	*Agarics bisporus*	Natural	Galβ1-3GalNAc (T), GlcNAc
27	LEL	*Lycopersicon esculentum*	Natural	Polylactosamine, (GlcNAc)n
28	STL	*Solanum tuberosum*	Natural	Polylactosamine, (GlcNAc)n
29	UDA	*Urtica dioica*	Natural	(GlcNAc)n
30	PWM	*Phytolacca americana*	Natural	(GlcNAc)n
31	Jacalin	*Artocarpus integrifolia*	Natural	Galβ1-3GalNAc (T), GalNAcα (Tn)
32	PNA	*Arachis hypogaea*	Natural	Galβ1-3GalNAc (T)
33	WFA	*Wisteria floribunda*	Natural	Terminal GalNAc, LacDiNAc
34	ACA	*Amaranthus caudatus*	Natural	Galβ1-3GalNAc (T)
35	MPA	*Maclura pomifera*	Natural	Galβ1-3GalNAc (T), GalNAca (Tn)
36	HPA	*Helix pomatia*	Natural	αGalNAc (A, Tn)
37	VVA	*Vicia villosa*	Natural	α,βGalNAc (A, Tn, LacDiNAc)
38	DBA	*Dolichos biflorus*	Natural	α,βGalNAc (A, Tn, LacDiNAc)
39	SBA	*Glycine max*	Natural	α,βGalNAc (A, Tn, LacDiNAc)
40	Calsepa	*Calystegia sepium*	Natural	Biantenna with bisecting GlcNAc
41	PTL I	*Psophocarpus tetragonolobus*	Natural	αGalNAc (A, Tn)
42	MAH	*Maackia amurensis*	Natural	α2-3Sia
43	WGA	*Triticum vulgaris*	Natural	(GlcNAc)n, polySia
44	GSLIA4	*Griffonia simplicifolia*	Natural	αGalNAc (A, Tn)
45	GSLIB4	*Griffonia simplicifolia*	Natural	αGal (B)

[1]Abbreviations: Gal (D-galactose), GalNAc (N-acetyl-galactosamine), GlcNAc (*N*-acetylglucosamine), Fuc (L-fucose), Glc (D-glucose), Sia (Sialic acid), LacNAc (N-acetyl-lactosamine).

[2]Specificity data was obtained by frontal affinity chromatography and glycoconjugate microarray.

Supplementary Table 6. A list of plasma markers measured in this study.

Category	Marker	Name	Method of Measurement
**Microbial translocation markers**	Zonulin	haptoglobin 2 precursor	ELISA
	LBP	Lipopolysaccharide binding protein	ELISA
	β-D-glucan	β-D-glucan	Limulus Amebocyte Lysate (LAL) assay
	sCD14	Soulble CD14	ELISA
	sCD163	Soulble CD163	ELISA
	OCLN	Occludin	ELISA
	I-FABP	Intestinal fatty-acid binding protein	ELISA
	Reg3A	Regenerating Family Member 3 Alpha	ELISA
**Inflammation and immune function markers**	IL-6	Interleukin 6	Multiplex meso scale cytokine assay
	TNF-α	tumor necrosis factor alpha	Multiplex meso scale cytokine assay
	GM-CSF	Granulocyte-macrophage colony-stimulating factor	Multiplex meso scale cytokine assay
	IFN-α2a	interferon α2a	Multiplex meso scale cytokine assay
	IFN-β	Interferon beta	Multiplex meso scale cytokine assay
	IFN-γ	Interferon gamma	Multiplex meso scale cytokine assay
	IL-1β	Interleukin 1β	Multiplex meso scale cytokine assay
	IL-2	Interleukin 2	Multiplex meso scale cytokine assay
	IL-4	Interleukin 4	Multiplex meso scale cytokine assay
	IL-10	Interleukin 10	Multiplex meso scale cytokine assay
	IL-12p70	Interleukin 12 p70	Multiplex meso scale cytokine assay
	IL-12/IL-23p40	Interleukin 12 p70	Multiplex meso scale cytokine assay
	IL-13	Interleukin 13	Multiplex meso scale cytokine assay
	IL-15	Interleukin-12/interleukin 23 p40	Multiplex meso scale cytokine assay
	IL-21	Interleukin 21	Multiplex meso scale cytokine assay
	IL-22	Interleukin 22	Multiplex meso scale cytokine assay
	IL-23	Interleukin 23	Multiplex meso scale cytokine assay
	IL-33	Interleukin 33	Multiplex meso scale cytokine assay
	Fractalkine	chemokine (C-X3-C motif) ligand 1 (CX3CL1)	Multiplex meso scale cytokine assay
	IP-10	C-X-C motif chemokine ligand 10 (CXCL10)	Multiplex meso scale cytokine assay
	MCP-2	Chemokine (C-C motif) ligand 8 (CCL8)	Multiplex meso scale cytokine assay
	MIP-1α	Macrophage inflammatory protein alpha	Multiplex meso scale cytokine assay
	SDF-1a	stromal cell-derived factor 1 (SDF1) or C-X-C motif chemokine 12 (CXCL12)	Multiplex meso scale cytokine assay
	CRP	C-reactive protein	ELISA
	d-dimer	D-dimer	ELISA
	MPO	Neutrophil myeloperoxidase	ELISA
	GDF-15	Growth/differentiation factor 15	ELISA
	C3a	Complement component 3a	ELISA
	Gal-1	Galectin-1	ELISA
	Gal-3	Galectin-3	ELISA
	Gal-9	Galectin-9	ELISA
**IgG *N*-glycome**	22 individual glycans structures categorized into 9 groups (Supplementary Table 4)		Capillary electrophoresis
**IgA total glycome**	Binding to 45 lectins with known glycan-binding specificity (Supplementary Table 5)		Lectin microarray
**Plasma *N*-glycome**	24 individual glycans structures categorized into 15 groups (Supplementary Table 3)		Capillary electrophoresis
**Plasma total glycome**	Binding to 45 lectins with known glycan-binding specificity (Supplementary Table 5)		Lectin microarray
**Plasma Metabolome**	278 plasma metabolites and three derivative ratios		Mass spectrometry
**Plasma Lipidome**	2015 plasma lipids categorized into 24 lipid classes		Mass spectrometry

Supplementary Table 7 . Top 50 metabolic pathways disrupted by severe COVID-19.

Pathway	*P* value	*FDR*	State	Z	Ratio
tRNA Charging	7.9433E-27	2.5119E-24	Activated	2.828	0.22
Superpathway of Citrulline Metabolism	3.9811E-16	7.9433E-14		1.89	0.256
Citrulline Biosynthesis	7.9433E-16	1E-13		1.134	0.333
Arginine Biosynthesis IV	3.9811E-14	3.1623E-12		0	0.333
Purine Nucleotides De Novo Biosynthesis II	6.3096E-14	3.9811E-12	Activated	2.121	0.22
Superpathway of Methionine Degradation	1.2589E-11	6.9183E-10		0	0.127
NAD biosynthesis II (from tryptophan)	3.1623E-11	1.6982E-09		0.816	0.226
Asparagine Biosynthesis I	5.0119E-11	2.3442E-09		0.447	0.625
Proline Biosynthesis II (from Arginine)	6.3096E-11	2.3988E-09		0	0.333
Phenylalanine Degradation IV (Mammalian, via Side Chain)	6.3096E-11	2.3988E-09		0.378	0.206
Purine Nucleotides Degradation II (Aerobic)	7.9433E-11	2.7542E-09			0.2
Urea Cycle	1.3804E-10	3.9811E-09			0.3
Glycolysis I	3.3884E-10	9.1201E-09			0.167
L-glutamine Biosynthesis II (tRNA-dependent)	4.4668E-10	1.0965E-08		1	0.455
Sirtuin Signaling Pathway	6.6069E-10	1.4454E-08		0.378	0.0401
Lysine Degradation V	6.166E-10	1.4454E-08		0.447	0.24
Gluconeogenesis I	7.7625E-10	1.5849E-08			0.149
Alanine Degradation III	3.7154E-09	6.4565E-08		0	0.667
Alanine Biosynthesis II	3.7154E-09	6.4565E-08		0	0.667
Arginine Degradation VI (Arginase 2 Pathway)	4.0738E-09	6.4565E-08		0	0.312
4-hydroxybenzoate Biosynthesis	4.0738E-09	6.4565E-08		0.447	0.312
NAD Biosynthesis from 2-amino-3-carboxymuconate Semialdehyde	4.0738E-09	6.4565E-08		1	0.312
Folate Polyglutamylation	7.9433E-09	1.1482E-07		-0.447	0.278
5-aminoimidazole Ribonucleotide Biosynthesis I	7.9433E-09	1.1482E-07		1.342	0.278
Glutamine Biosynthesis I	8.7096E-09	1.2023E-07		-1	0.571
Citrulline Degradation	1.7378E-08	2.2909E-07		0	0.5
(S)-reticuline Biosynthesis II	3.0903E-08	3.8019E-07		1	0.444
Glycine Degradation (Creatine Biosynthesis)	3.0903E-08	3.8019E-07		1	0.444
γ-glutamyl Cycle	3.8905E-08	4.6774E-07			0.208
4-aminobutyrate Degradation I	5.1286E-08	5.8884E-07		0	0.4
Glutathione Biosynthesis	8.1283E-08	8.9125E-07		0	0.364
Folate Transformations I	8.9125E-08	9.5499E-07			0.179
Glutamate Degradation III (via 4-aminobutyrate)	1.2023E-07	1.2589E-06			0.333
L-carnitine Biosynthesis	1.7378E-07	1.7378E-06		-1	0.308
Arginine Degradation I (Arginase Pathway)	1.7378E-07	1.7378E-06			0.308
PFKFB4 Signaling Pathway	1.9055E-07	1.8197E-06	Activated	2	0.0968
Proline Biosynthesis I	2.4547E-07	2.2909E-06		-1	0.286
Cysteine Biosynthesis III (mammalia)	3.8019E-07	3.4674E-06			0.135
Salvage Pathways of Pyrimidine Ribonucleotides	4.3652E-07	3.7154E-06		0	0.0609
Selenocysteine Biosynthesis II (Archaea and Eukaryotes)	4.3652E-07	3.7154E-06		1	0.25
Citrulline-Nitric Oxide Cycle	4.3652E-07	3.7154E-06			0.25
Phosphatidylcholine Biosynthesis I	5.7544E-07	4.5709E-06		1	0.235
CMP-N-acetylneuraminate Biosynthesis I (Eukaryotes)	5.7544E-07	4.5709E-06			0.235
Adenine and Adenosine Salvage VI	6.3096E-07	4.8978E-06			0.6
4-hydroxyphenylpyruvate Biosynthesis	6.3096E-07	4.8978E-06			0.6
TCA Cycle II (Eukaryotic)	7.4131E-07	5.4954E-06			0.119
Uridine-5'-phosphate Biosynthesis	9.3325E-07	6.4565E-06		1	0.211
Superpathway of Serine and Glycine Biosynthesis I	9.3325E-07	6.4565E-06			0.211
Sucrose Degradation V (Mammalian)	9.3325E-07	6.4565E-06			0.211

Supplementary Table 8. List of the gut-associated and gut microbiota-associated metabolites detected in our study using untargeted LC-MS/MS (50 of the 278 metabolites identified in plasma).

Name	Reference
α-Hydroxyhippuric acid	(1)
β-D-Glucopyranuronic acid	(2)
(2R)-2,3-Dihydroxypropanoic acid	(3)
16-Hydroxyhexadecanoic acid	(4)
2-Hydroxycinnamic acid	(5)
2-Hydroxyhippuric acid	(1)
2-Hydroxyvaleric acid	(6)
2,3-Dihydroxybenzoic acid	(1)
2,4-Dihydroxybenzoic acid	(7)
3-Hydroxybutyric acid	(8)
3-Indoxyl sulphate	(9)
3-methylphenylacetic acid	(7)
4-Hydroxybenzaldehyde	(7)
4-Hydroxyproline	(10)
Acetylcholine	(11)
Allantoin	(12)
Cholic acid	(13)
Choline	(14)
Citrulline	(15)
D-(-)-Mannitol	(16)
D-Glucose	(17)
Decanoic acid	(18)
Deoxycholic Acid	(19)
Glucose 6-phosphate	(20)
Glycine	(21)
Glycocholic acid	(19)
Glycoursodeoxycholic acid	(19)
Glycyl-L-leucine	(22)
Hippuric acid	(1, 7)
Indole-3-acetic acid	(23)
Indole-3-lactic acid	(23)
Indole-3-pyruvic acid	(19)
Kynurenic acid	(24)
L-Isoleucine	(23)
L-Kynurenine	(75)
L-Lactic acid	(24, 25)
L-Leucine	(24)
L-Serine	(24, 25)
L-Threonine	(25)
L-Tryptophan	(23, 24)
L-Valine	(24)
N-Acetyl-DL-tryptophan	(26)
Pentadecanoic acid	(18)
Phosphoenolpyruvic acid	(24)
Pipecolic acid	(25)
Pyruvic acid	(24)
Succinic acid	(27)
Taurochenodeoxycholic Acid	(19)
Trans-Cinnamic acid	(28)
Trimethylamine N-oxide	(29)

Supplementary Table 9. Two thousand fifteen lipids identified in this study were assigned to 24 lipid classes.

Group	Abbreviation	Class	Number of lipids
Phospholipids	CL	Cardiolipin	2
	LPA	Lysophosphatidic acid	2
	PA	Phosphatidic acid	8
	LPC	Lysophosphatidylcholine	110
	PC	Phosphatidylcholine	262
	LPE	Lysophosphatidylethanolamine	22
	PE	Phosphatidylethanolamine	139
	PG	Phosphatidylglycerol	10
	LPI	Lysophosphatidylinositol	6
	PI	Phosphatidylinositol	47
	LPS	Lysophosphastidylserine	1
	PS	Phosphatidylserine	11
Neutral lipids	ChE	Cholesterol ester	22
	DG/DAG	Diglyceride	58
	TG/TAG	Triglyceride	742
Sphingolipids	Cer	Ceramide	121
	Hex1Cer	Simple Glc series (Ceramide with 1 hexose)	21
	Hex2Cer	Simple Glc series (Ceramide with 2 hexose)	10
	Hex3Cer	Simple Glc series (Ceramide with 3 hexose)	9
	LSM	Lysosphingomyelin	1
	SM	Sphingomyelin	359
	SPH	Sphingosine	1
Other lipids	AcCa	Acyl carnitine	46
	Co	Coenzyme	5

Supplementary Table 10. Demographics, clinical data, and levels of selected measures in the moderate and severe groups of the main study cohort.

ID	Group	Age (years)	Sex	Ethinicity	Deceased	Body mass index	Diabetes	High blood pressure	Asthma	Hydroxychloroquine	Remdesivir	Tocilizumab	Steroids	Antibiotic	IL-6 (pg/ml)	Zonulin (ng/ml)	LBP (ng/ml)	βglucan (pg/ml)	sCD14 (ng/ml)	Citrulline (relative levels)	L-Kynurenine/L-Tryptophan
1	Moderate	57	Female	African American	Yes	>25	No	No	No	Yes	No	No	No	Yes	1763.257647	193.8942	9304.6	17.83	3493.879433	1821755426	0.318309463
2	Moderate	53	Male	Caucasion	No	>25	No	No	No	Yes	No	No	No	No	18.01473445	49.6164	21917.376	26.72	3455.754172	718381364.6	0.070994515
5	Moderate	58	Female	African American	No	<25	No	No	No	No	No	No	Yes	Yes	21.68378376	40.9002	16070.458	42.704	2370.889564	712152121.3	0.049167598
8	Moderate	61	Female	African American	No	>25	Yes	Yes	No	No	No	No	Yes	No	6.349896414	32.7362	32909.685	52.786	2950.155691	787957761.3	0.073773424
9	Moderate	61	Female	African American	No	>25	Pre	Yes	No	Yes	No	No	No	Yes	14.72774328	25.4352	27308.872	49.936	3870.557475	1081500040	0.100074232
10	Moderate	58	Female	Hispanic	No	>25	No	No	No	Yes	No	No	No	Yes	33.61113291	77.483	43753.549	73.084	3946.763302	650814955.9	0.253576756
12	Moderate	62	Male	Caucasion	No	>25	Pre	Yes	No	Yes	No	No	No	Yes	2.154274243	47.1282	23806.672	64.42	1667.576587	868575465.6	0.068367184
13	Moderate	56	Male	African American	No	>25	No	No	No	No	No	No	No	No	10.22549033	12.7694	22474.136	55.522	3639.788518	694690536.8	0.066452871
15	Moderate	62	Male	Caucasion	No	<25	Yes	Yes	No	No	No	No	No	No	3.10147469	0.5856	18370.92	51.33	2204.235012	2396648670	0.088245657
16	Moderate	54	Male	African American	No	>25	No	No	No	Yes	No	No	No	No	8.36005405	56.8008	32301.397	62.37	1877.763692	510506740.2	0.035193984
17	Moderate	57	Female	Caucasion	No	>25	No	Yes	Yes	No	No	No	Yes	Yes	1.901578289	0.5856	18912.9	19.88	2027.288127	782203371.7	0.03068764
26	Moderate	63	Male	Hispanic	No	<25	Yes	Yes	No	No	No	No	Yes	Yes	220.9539105	54.7636	39661.744	59.528	3365.222049	814348893.5	0.081288883
27	Moderate	57	Male	Hispanic	No	<25	Yes	Yes	No	No	No	No	Yes	Yes	1.078827252	5.0796	7822.961	30.788	1855.327767	1262606529	0.019510952
31	Moderate	59	Female	African American	Yes	>25	Yes	Yes	Yes	Yes	No	Yes	Yes	Yes	109.2382182	123.3182	65613.144	80.456	4690.125491	806015750.1	0.328378664
34	Moderate	58	Male	Hispanic	No	>25	Pre	Yes	No	No	No	No	Yes	Yes	4.747457275	65.789	18899.985	32.41	1954.250577	1521058972	0.360965105
36	Moderate	59	Female	Hispanic	No	>25	No	Yes	Yes	No	No	No	No	No	50.99140744	186.2356	39888.445	60.264	2521.605962	457646499.1	0.144641071
37	Moderate	59	Female	Hispanic	No	<25	Yes	Yes	No	Yes	No	No	No	Yes	13.42164849	12.3572	41379.358	55.048	3079.004266	174336612.7	0.069977164
38	Moderate	62	Male	African American	No	>25	No	No	No	No	No	No	No	No	13.99319259	89.247	19775.875	62.86	2181.267201	550382249.4	0.049178512
39	Moderate	64	Female	African American	No	>25	No	Yes	No	No	No	No	Yes	Yes	1.429866392	27.0292	19278.399	49.052	2152.988148	1005791769	0.145485036
40	Moderate	51	Female	Unknown	No	>25	No	No	No	No	Yes	No	No	No	1.646799114	30.3726	6096.596	35.732	1393.232256	579645991.7	0.031223194
3	Severe	53	Male	Hispanic	No	>25	No	No	No	Yes	No	No	Yes	Yes	30.1633883	58.659	45712.063	82.202	2513.57029	441228129.5	0.08463056
4	Severe	57	Female	African American	No	>25	Pre	No	No	Yes	Yes	No	Yes	Yes	619.0606777	34.6646	45439.47	52.812	3624.039042	514633656.5	0.241071742
6	Severe	63	Male	African American	No	>25	Yes	Yes	No	Yes	No	No	No	No	3.573688648	30.3104	25787.79	41.318	2430.854067	875668616.1	0.097722934
7	Severe	57	Male	African American	No	>25	Yes	Yes	No	Yes	No	Yes	Yes	Yes	516.9583792	100.6376	56150.038	101.648	4690.125491	984414695.3	0.410516182
11	Severe	63	Female	African American	Yes	<25	Yes	Yes	No	No	No	No	Yes	No	6.85243542	89.3092	19488.006	52.076	2315.330963	659380099.4	0.058170928
14	Severe	59	Male	Hispanic	No	>25	Pre	No	No	Yes	No	No	No	Yes	10.27798698	32.433	30034.297	70.83	2679.447992	813006537.7	0.069817304
18	Severe	54	Male	African American	No	>25	Yes	No	Yes	No	Yes	No	No	Yes	20.88394583	0.5856	25197.66	47.174	3306.518939	561860632	0.110848595
19	Severe	52	Male	Hispanic	Yes	>25	Yes	Yes	No	Yes	No	Yes	Yes	Yes	118.1545736	101.7884	44794.181	60.292	4396.293347	611287349.4	0.117013349
20	Severe	53	Male	Caucasion	No	>25	Yes	No	No	Yes	No	Yes	Yes	Yes	24.14887075	165.911	50243.932	44.938	3663.26299	629935512.5	0.148348286
21	Severe	56	Female	Hispanic	No	>25	Yes	Yes	No	No	Yes	No	No	No	23.41418214	47.1516	38649.275	70.882	3477.845967	359297583.8	0.053024607
22	Severe	52	Male	Other	No	>25	No	Yes	No	No	No	No	No	Yes	71.96666096	9.0916	41002.193	39.906	3346.3174	722847555	0.081676354
23	Severe	63	Male	African American	Yes	>25	No	No	No	No	No	Yes	Yes	Yes	20.28186358	46.8406	54026.01	72.846	3758.345672	625376939.2	0.109617984
24	Severe	63	Female	Hispanic	Yes	>25	Pre	Yes	No	Yes	No	No	Yes	Yes	225.3700541	52.6488	63356.428	60.406	1952.345076	289965314.9	0.265135673
25	Severe	63	Female	Caucasion	Yes	>25	Yes	Yes	No	Yes	No	Yes	Yes	Yes	265.4670538	177.1618	35176.898	79.79	4690.125491	577143302.6	0.427586715
28	Severe	59	Female	Hispanic	No	>25	Pre	Yes	No	Yes	No	No	No	Yes	125.2595669	29.183	56041.668	98.72	4395.229986	388229892.3	0.205720859
29	Severe	63	Male	Caucasion	No	>25	Yes	Yes	No	No	No	No	No	Yes	63.18794679	70.2674	31012.407	50.664	4690.125491	1012993979	0.233867928
30	Severe	64	Male	African American	Yes	>25	Yes	Yes	No	No	No	No	No	Yes	11.06562902	36.173	16195.838	42.738	1515.144719	615942637.8	0.032801245
32	Severe	59	Female	Hispanic	No	>25	Pre	Yes	No	Yes	Yes	Yes	No	Yes	1.754595678	9.496	15714.848	47.298	1817.708363	1105465401	0.065271545
33	Severe	56	Male	Hispanic	No	>25	Yes	No	No	No	No	No	Yes	Yes	14.66199276	4.512	30378.645	45.368	2431.994146	663946437.6	0.058912279
35	Severe	51	Male	Hispanic	No	>25	Pre	No	No	No	Yes	No	No	Yes	132.1470257	67.0486	68888.237	65.192	2759.626511	292532690.6	0.231106412

References

1. Gonthier MP, Cheynier V, Donovan JL, Manach C, Morand C, Mila I, et al. 2003. Microbial aromatic acid metabolites formed in the gut account for a major fraction of the polyphenols excreted in urine of rats fed red wine polyphenols. J Nutr 133:461-7.
2. Pellock SJ, Redinbo MR. 2017. Glucuronides in the gut: Sugar-driven symbioses between microbe and host. J Biol Chem 292:8569-8576.
3. Monagas M, Urpi-Sarda M, Sanchez-Patan F, Llorach R, Garrido I, Gomez-Cordoves C, et al. 2010. Insights into the metabolism and microbial biotransformation of dietary flavan-3-ols and the bioactivity of their metabolites. Food Funct 1:233-53.
4. Zhitao H, Ailin S, Qingshuang W, Changyuan H, Liying S, Xianming S, et al. 2020. Research Square doi:10.21203/rs.3.rs-70833/v1.
5. Coman V, Vodnar DC. 2020. Hydroxycinnamic acids and human health: recent advances. J Sci Food Agric 100:483-499.
6. Skrzypecki J, Nieweglowska K, Samborowska E. 2020. Valeric Acid, a Gut Microbiota Product, Penetrates to the Eye and Lowers Intraocular Pressure in Rats. Nutrients 12.
7. Marhuenda-Munoz M, Laveriano-Santos EP, Tresserra-Rimbau A, Lamuela-Raventos RM, Martinez-Huelamo M, Vallverdu-Queralt A. 2019. Microbial Phenolic Metabolites: Which Molecules Actually Have an Effect on Human Health? Nutrients 11.
8. Selkrig J, Wong P, Zhang X, Pettersson S. 2014. Metabolic tinkering by the gut microbiome: Implications for brain development and function. Gut Microbes 5:369-80.
9. Farowski F, Els G, Tsakmaklis A, Higgins PG, Kahlert CR, Stein-Thoeringer CK, et al. 2019. Assessment of urinary 3-indoxyl sulfate as a marker for gut microbiota diversity and abundance of Clostridiales. Gut Microbes 10:133-141.
10. Huang YY, Martinez-Del Campo A, Balskus EP. 2018. Anaerobic 4-hydroxyproline utilization: Discovery of a new glycyl radical enzyme in the human gut microbiome uncovers a widespread microbial metabolic activity. Gut Microbes 9:437-451.
11. Rea K, Dinan TG, Cryan JF. 2016. The microbiome: A key regulator of stress and neuroinflammation. Neurobiol Stress 4:23-33.
12. Pan L, Han P, Ma S, Peng R, Wang C, Kong W, et al. 2020. Abnormal metabolism of gut microbiota reveals the possible molecular mechanism of nephropathy induced by hyperuricemia. Acta Pharm Sin B 10:249-261.
13. Molinero N, Ruiz L, Sanchez B, Margolles A, Delgado S. 2019. Intestinal Bacteria Interplay With Bile and Cholesterol Metabolism: Implications on Host Physiology. Front Physiol 10:185.
14. Arias N, Arboleya S, Allison J, Kaliszewska A, Higarza SG, Gueimonde M, et al. 2020. The Relationship between Choline Bioavailability from Diet, Intestinal Microbiota Composition, and Its Modulation of Human Diseases. Nutrients 12.
15. Fragkos KC, Forbes A. 2018. Citrulline as a marker of intestinal function and absorption in clinical settings: A systematic review and meta-analysis. United European Gastroenterol J 6:181-191.
16. Maekawa M, Maekawa M, Ushida K, Hoshi S, Kashima N, Ajisaka K, et al. 2005. Butyrate and propionate production from D-mannitol in the large intestine of pig and rat. Microbial Ecology in Health and Disease 17:169-176.
17. Utzschneider KM, Kratz M, Damman CJ, Hullar M. 2016. Mechanisms Linking the Gut Microbiome and Glucose Metabolism. J Clin Endocrinol Metab 101:1445-54.
18. Mathewson ND, Jenq R, Mathew AV, Koenigsknecht M, Hanash A, Toubai T, et al. 2016. Gut microbiome-derived metabolites modulate intestinal epithelial cell damage and mitigate graft-versus-host disease. Nat Immunol 17:505-513.
19. Ridlon JM, Kang DJ, Hylemon PB, Bajaj JS. 2014. Bile acids and the gut microbiome. Curr Opin Gastroenterol 30:332-8.
20. Di Rienzi SC, Britton RA. 2020. Adaptation of the Gut Microbiota to Modern Dietary Sugars and Sweeteners. Adv Nutr 11:616-629.

21. Craft IL, Geddes D, Hyde CW, Wise IJ, Matthews DM. 1968. Absorption and malabsorption of glycine and glycine peptides in man. Gut 9:425-37.
22. Yasumoto K, Sugiyama K. 1980. Perturbation by Bestatin of Glycyl-l-leucine Absorption in Isolated Epithelial Cells from Rat Intestine. Agricultural and Biological Chemistry 44:1339-1344.
23. Gao J, Xu K, Liu H, Liu G, Bai M, Peng C, et al. 2018. Impact of the Gut Microbiota on Intestinal Immunity Mediated by Tryptophan Metabolism. Front Cell Infect Microbiol 8:13.
24. Krautkramer KA, Fan J, Backhed F. 2020. Gut microbial metabolites as multi-kingdom intermediates. Nat Rev Microbiol doi:10.1038/s41579-020-0438-4.
25. Jain A, Li XH, Chen WN. 2019. An untargeted fecal and urine metabolomics analysis of the interplay between the gut microbiome, diet and human metabolism in Indian and Chinese adults. Sci Rep 9:9191.
26. Zhang L, Ouyang Y, Li H, Shen L, Ni Y, Fang Q, et al. 2019. Metabolic phenotypes and the gut microbiota in response to dietary resistant starch type 2 in normal-weight subjects: a randomized crossover trial. Sci Rep 9:4736.
27. Oliphant K, Allen-Vercoe E. 2019. Macronutrient metabolism by the human gut microbiome: major fermentation by-products and their impact on host health. Microbiome 7:91.
28. Kong J-Q. 2015. Phenylalanine ammonia-lyase, a key component used for phenylpropanoids production by metabolic engineering. RSC Advances 5:62587-62603.
29. Heianza Y, Ma W, DiDonato JA, Sun Q, Rimm EB, Hu FB, et al. 2020. Long-Term Changes in Gut Microbial Metabolite Trimethylamine N-Oxide and Coronary Heart Disease Risk. J Am Coll Cardiol 75:763-772.
